# Postdigital Bystanding: Youth Experiences of Sexual Violence Workshops in Schools in England, Ireland, and Canada

**DOI:** 10.3390/bs15010081

**Published:** 2025-01-18

**Authors:** Jessica Ringrose, Debbie Ging, Faye Mishna, Betsy Milne, Tanya Horeck, Kaitlynn Mendes

**Affiliations:** 1 IOE, UCL’s Faculty of Education and Society, University College London, London WC1H 0AL, UK; 2 School of Communications, Dublin City University, D09 E432 Dublin, Ireland; debbie.ging@dcu.ie; 3 Department of Social Work, University of Toronto, Toronto, ON M5S 1A1, Canada; f.mishna@utoronto.ca; 4 Centre of Multidisciplinary and Intercultural Inquiry, University College London, London WC1E 6BT, UK; uclziln@ucl.ac.uk; 5 Cambridge School of Creative Industries, Anglia Ruskin University, Cambridge CB1 1PT, UK; tanya.horeck@aru.ac.uk; 6 Department of Sociology, Western University, London, ON N6A 3K7, Canada; kaitlynn.mendes@uwo.ca

**Keywords:** bystanding, postdigital, school, sexual violence, intersectionality, defensive masculinity, sexism, elitism, racism

## Abstract

In this paper, we report on creative- and arts-based sexual violence and bystander intervention workshops we developed and researched in England, Ireland, and Canada, through evaluation surveys, observations, and focus group interviews with nearly 1200 young people (aged 13–18). Whist the young people generally reported benefitting from the intervention, in the context of increasing use of digital technologies amongst youth, we explore the context-specific challenges they faced in learning about and being supported through bystander strategies across a wide range of diverse school spaces. We use the term postdigital bystanding to explicitly explore how teen’s digital networks are often connected to the school-based ‘real life’ peer group, in ways that complicate clear distinctions between online and offline, arguing that these postdigital dynamics have not yet been adequately considered in bystanding interventions. We analyse how the intersectional community, cultural, and identity-specific factors in particular schooling environments shape responses to bystanding in postdigital environments, including how factors of sexism, defensive masculinity, elitism, racism, and a reluctance to report digital issues played out in the responses to the workshops. Finally, following young people’s suggestions, we recommend that schools need to cultivate better safety and support strategies for youth in order to make postdigital bystander interventions more responsive and therefore effective in challenging and preventing sexual violence in society.

## 1. Introduction: Gaps in Sexual Violence and Bystander Interventions

There is a wealth of literature on preventative interventions for youth sexual- and gender-based violence (SGBV). According to key systematic reviews, youth violence interventions vary according to their aims, approaches, activities, length, target audiences, and facilitator training (Kovalenko et al., 2022). Many interventions rely on a bystander model which aims to move beyond the traditional focus of violence prevention programmes in the victim/perpetrator dichotomy and consider how bystanders shape interactions and may play a role in violence prevention (Brown et al., 2014; Butler et al., 2024; Coker et al., 2020; Kettrey & Marx, 2019). For instance, peer mentoring programmes, such as ‘mentors in violence’, use bystanding perspectives to train individuals to recognise violence and support victims to recognise and challenge gendered and sexualised norms (Bruno et al., 2020). Bystander interventions seek to improve our understanding of gender inequitable attitudes and deconstruct rape myths that perpetuate sexual violence (Finnie et al., 2022; Lundgren & Amin, 2015; Mujal et al., 2021). However, while there are some reported increases in bystander actions, the degree of success of interventions varies considerably (Crooks et al., 2019). In order to think about what works or not, there are several research gaps on bystander interventions into sexual violence, which we aim to address in this paper.

First, a large portion of the bystander prevention literature focuses on interventions in college and university settings (Powers & Leili, 2018; Hines & Reed, 2015; Brown et al., 2014; Kettrey & Marx, 2019; Kovalenko et al., 2022). A limited number have examined secondary school-based lessons or programmes or ways to integrate these topics into the existing curriculum to enable systematic delivery (De La Rue et al., 2017; Lundgren & Amin, 2015). In the US, scholars have argued bystander approaches should be implemented as part of comprehensive sex education (Schneider & Hirsch, 2020) and, in the US and England, as part of a ‘whole school approach’ (Storer et al., 2017; EVAW, 2023). School-based sex education has likewise been noted to pay inadequate attention to issues of sexual- and gender-based violence, demonstrating a lack of awareness regarding how to practically enable bystander interventions, which could potentially empower young people through an understanding of their rights (Setty & Dobson, 2023). In this paper, we respond to this specific gap, exploring in-depth experiences and reactions to bystander interventions in schooling environments.

Second, bystander research has, to date, failed to adequately consider school-related and environmental factors that influence or impede bystander behaviours in the context of youth gender and sexual violence, sometimes known as ‘teen dating violence’ (Storer et al., 2017). Storer et al. (2017) outline three significant ways that a school environment can create barriers that undermine active bystander intervention work, including the following: ineffective school-based responses to sexual violence; lack of attention paid to dating violence; and gender inequitable schooling environments. Poor understanding of the issues on the part of schools leads to inappropriate and ineffective responses, such as relativizing dating violence as simply another form of (cyber)bullying, or responding with detentions or suspensions, which fail to address the root causes or drivers of the behaviour. For instance, these authors (Storer et al., 2017, p. 92) discussed how students across their study endorsed and condoned ‘slut shaming’, noting one participant who said ‘Guys don’t just call girls sluts for no reason’. These ‘assumptions’ meant some students could not recognise sexism as ‘intervention worthy’ (Storer et al., 2017, p. 92). We will address how school environments shape the uptake of bystander interventions, for instance, how sexist assumptions and sexual double standards are held not only by students, but are part of wider school cultures, including being condoned by some teachers, showing how this impedes bystander work. We draw upon a sociological and intersectional lens (Collins, 2015) that is attentive to the play of power and privilege in the diverse school sites in our research. In particular, we look at how issues of gender and sexism are shaped through intersections with fee-paying class privilege and race dynamics across diverse school sites (Phoenix, 2008).

Third, the majority of bystanding research has not accounted for how digital and social media contexts now complicate what it means to be a bystander. The question of how to respond to digitally mediated experiences of gender- and sexual-based violence in and around schools is paramount, but has barely been considered in the bystander research. Sidsel Harder’s (2020) work is an exception. Harder has addressed some of these gaps by pointing to the need to better understand bystanding in relation to youth digital intimacies and sexual cultures online and in social media contexts. Taking a qualitative approach to understanding the emotional process of witnessing image-based abuse online, Harder argues there is a need for more complexity in bystander interventions around digital sexual violence, acknowledging ‘ambivalence’ on the part of young people, whereby ‘bystanders find such situations quite difficult to manage’ (Harder, 2020, p. 666). Harder’s approach grapples with the ‘emotional complexities’ of young people’s reactions, including uncertainty of what to do, how to report and support peers, and worry about placing themselves at risk by openly challenging sexual violence on their screens, devices, and apps. As Harder concludes (Harder, 2020, p. 666), ‘future bystander-focused interventions should seek to incorporate these complexities rather than simply advising young people to “speak up!”—because bystanders already know that they should act, but especially men seldom feel able to’. We agree with Harder’s call to attend to emotional complexities and the role of the digital in relation to bystanding, seeking to apply these understandings to youth and school settings. In the contemporary digitally mediated youth culture, there is an urgent need to better understand how young people navigate the entanglement of online and offline experiences and find new ways to support and respond to these emotionally challenging bystanding experiences, particularly in and around schools.

To address the above gaps, we develop the term ‘postdigital bystanding’ to emphasise the fact that the online and offline are now thoroughly enmeshed and that incidents of abuse and sexual- and gender-based violence are constantly traversing digital and non-digital spaces (like school, street, and home). The concept of the ‘post-digital’ (Cramer, 2015) helps us to grapple with online/offline relationality and how the ‘non-digital’ space is thoroughly impacted by what happens in the digital realm and vice versa. ‘Post’, then, signals not that anything is over or finished, but that we are living with the consequences of the entanglement of the digital and analogue (Evans & Ringrose, 2024) in ways that have yet to be fully considered within bystander research. We argue there is a need to account for the dynamic and complex ways in which young people’s digital experiences overlap and are deeply intertwined with their ‘real life’ material worlds, including at school (Setty, 2023). Take, for instance, Finkelhor et al.’s (2021) recent review of image-based sexual abuse, which found significant overlap between young people’s online and offline experiences, as did Mitchell et al.’s (2016) study of online and offline peer sexual harassment among youth, which found that many experiences of harassment online were connected to peer relationships offline (Mitchell et al., 2016). We call these postdigital dynamics, arguing that these will shape the capacity for active bystanding in school, as we will explore.

## 2. Methodology: Co-Producing and Piloting Sexual Violence and Active Bystander Workshops

England, Ireland, and Canada have been slow to create educational policy and guidance on sexual violence prevention and particularly programming on supporting youth through intervention strategies. Both methodologically and pedagogically, there is an absence of accounting for young people’s (digital and sexual) rights when it comes to developing interventions on sexual harassment and abuse internationally (Albury, 2017). We thus partnered with an award-winning UK sex education charity to produce two evidence-based workshops for secondary school students. The workshops were developed over a 6-month period, and we also consulted with a major UK teachers association, asking experts to feedback on the developing workshops. The workshops are designed to be delivered by teachers or external agencies in relation to sex education curriculum, but can be adapted to any curriculum model and are presently being adapted into international school sites.

**Figure 1:**
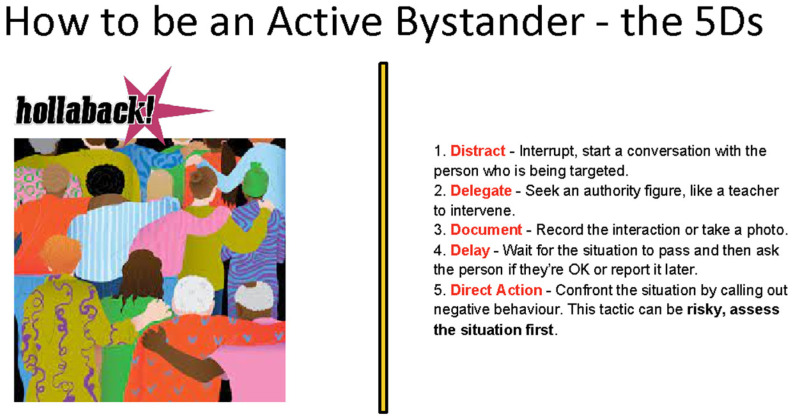
‘How to be an Active Bystander—the 5Ds’ slide.

Workshop One, which we have explored previously (Ging et al., 2024), educates students about sexual- and gender-based violence, including specific forms of technology-facilitated gender-based sexual violence. Workshop Two—the focus of this paper—introduces students to a 5D model of active bystanding developed by a sexual violence charity’s (Right To Be, formerly Hollaback). The 5D model includes the following: Distract, Delegate, Document, Delay, and Direct Action (Figure 1).

**Figure 2:**
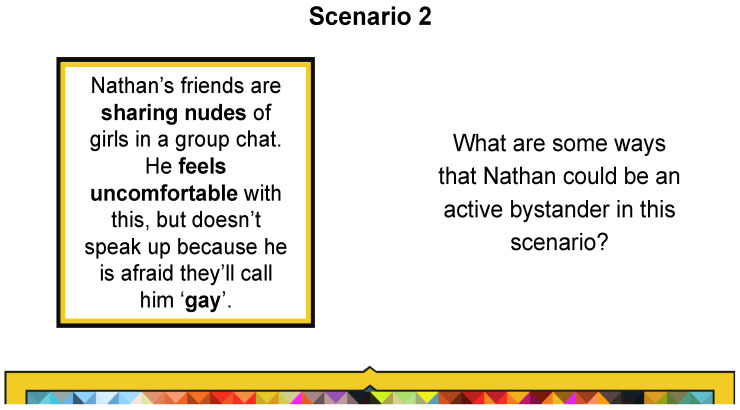
Scenario 2 slide.

There are various scenarios in the workshop to help students debate ways of enacting active bystanding and the 5Ds. For instance, Scenario Two considers a postdigital context of bystanding when nudes are non-consensually shared in a WhatsApp group (Figure 2).

**Figure 3:**
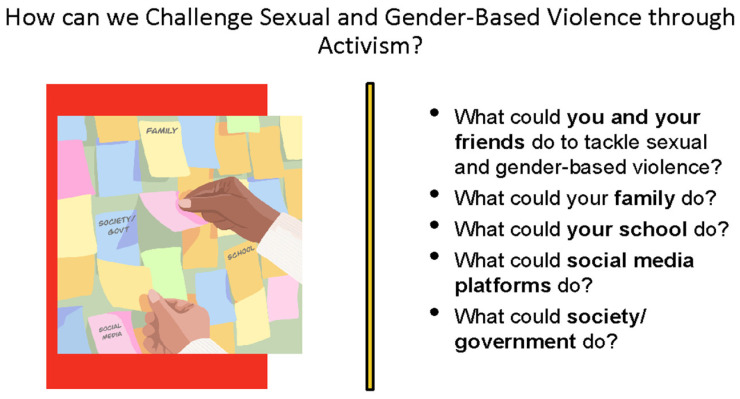
‘How can we Challenge Sexual and Gender-Based Violence’ post-it note activity slide.

Workshop Two also features an interactive post-it notes activity (Figure 3), which asks participants to suggest ideas about what social media, schools, families, and government could do to challenge sexual violence.

The workshop offers students digital defence strategies, such as how to report content and provides self-care strategies when using social media, including an arts-based pedagogy task where young people can create their own social media campaign to challenge everyday sexual violence. Across the study, we worked with schools’ leadership teams to address any disclosures from young people and offer extra support after workshops.

To study the delivery and reception of the workshop, we conducted observations, looking for how students engaged in the discussions and activities and how they interacted with the facilitator and peers. We administered pre- and post-evaluation surveys to gather feedback and measure knowledge before and after the workshops. Using arts-based methodologies (Renold, 2018), we collected written and drawn data produced by the young people during the workshops, including post-it notes from group activities as well as art activism drawings. Following the workshop observations, we conducted focus group interviews with a sub-sample of student participants and some teachers. Whilst the studies were connected, it was incumbent upon each research team to obtain ethical approval from their respective university ethics boards, given the sensitivity and cultural specificity of the educational intervention in school sites. The research followed strict ethical guidelines of gaining parental consent for interviews with young people under 16 years of age, and informed consent before the interviews from all participants. Interviews were digitally recorded and transcribed verbatim and anonymised. Pseudonyms are used throughout.

### Sample

In England, we piloted and observed 46 workshops in eight schools with approximately 1000 young people across England, including two schools in London comprising highly diverse and under-privileged student bodies. In all but one school, we worked with entire year groups, typically between 150 and 200 young people in year 9 (aged 13–14) or year 10 (aged 14–15), in classrooms of about 20–30 students. In total, 733 young people completed the pre- and post-survey evaluations. Participation in the workshops was compulsory as part of their regular RSE (Relationship and Sex Education) sessions in the schools, whereas participation in the focus groups following the workshops was optional based on volunteers and parental consent. We conducted 24 focus groups with 147 students to obtain their impressions and reflections on the workshops and any recommendations for improvement. We conducted two focus groups with 13 teachers. Ireland and Canada offered opportunities to do spin-off studies working with smaller numbers of young people, but which could still test the resource in their specific contexts.

In Ireland, we worked with 60 young people (aged 15–17) in two schools. The workshops were delivered by teachers who had been trained by the sex education facilitators. We then conducted 10 focus groups with student participants in groups of around 6, all of whom had completed the pre-and post-workshop evaluations. Participation was self-selecting, and there were considerably more girls than boys.

In Canada, we worked in one school with 108 students between 15 and 17 years; the workshops were condensed to a single longer 1.5 h delivery. The workshop was delivered five times by the school social workers, along with research team members who conducted observations. In total, 103 participants completed the pre- and post-workshop evaluations, and we conducted six follow up interviews. As was the case in Ireland, young people could opt out of the workshops, which skewed the attendance to those whose parents consented and who had interest in the topic, with more girls than boys.

Across the whole data set, interview transcription notes were placed into codes using NVivo and then thematically analysed (Braun & Clarke, 2006). Observations and written/visual data, including post-it notes, were manually coded and placed into themes. We used the pre- and post-workshop surveys, observations, focus groups, and workshop data to explore the young people’s perspectives on the workshops. Our analysis is not meant to be comparative, but rather to show how contextual factors shape the workshop experiences. Given the focus groups’ exploratory nature, we found that they led to larger conversations about young people’s critiques of, and ideas for, changing the school environment to better respond to sexual violence and support bystander intervention work.

## 3. Results

In this section, we report the key findings related to bystanding organised under three key thematic categories: (1) the positive impacts of learning about postdigital bystanding; (2) intersectional challenges of bystanding (a. sexism; b. masculine defensiveness; c. elitism; d. racism; e. reluctance to report digital issues); and (3) post-it notes for change: capturing young people’s ideas for making schools safer places.

### 3.1. Positive Impacts of Learning About Postdigital Bystanding

Through our pre- and post-workshop surveys, in Workshop One, we learned that young people had patchy understandings of sexual violence. When asked whether they had been taught about sexual violence previously, 55% of young people in England, 54% in Canada, and 69% in Ireland responded ‘no’ or ‘I don’t know’. Young people reported that their sex education did the ‘bare minimum’, and they did not learn about issues like ‘sexual harassment online’. They noted receiving mostly abstinence-only approaches to sex education, particularly around digital issues like sexing and nudes, through messages such as ‘nudes are illegal’ and ‘don’t do it until you’re 16’ (South West England), rather than education that addressed their rights and their experiences of digital sexual harms and abuse.

The second workshop in England and Ireland and the second half of the workshop in Canada address these educational gaps by introducing young people to the concept of active bystanding in relation to sexual violence (online and offline). The post-workshop survey confirmed that young people increased their understandings of what it meant to be an active bystander across all three countries, from 58% before to 98% in England, from 65% to 98% in Ireland, and 63% to 90% in Canada. Positive responses included the following:

Cali: Strategies for the bystanders to harassment or abuse, I think I learned a lot from that!.

(Ontario, Canada)

Jenny: I’m more open to speaking about [sexual violence] as well.

(South Dublin, Ireland)

Robin: Like yesterday I found myself telling people what you guys were saying in the workshop and just teaching them about it as well.

(Cornwall, UK)

**Figure 4:**
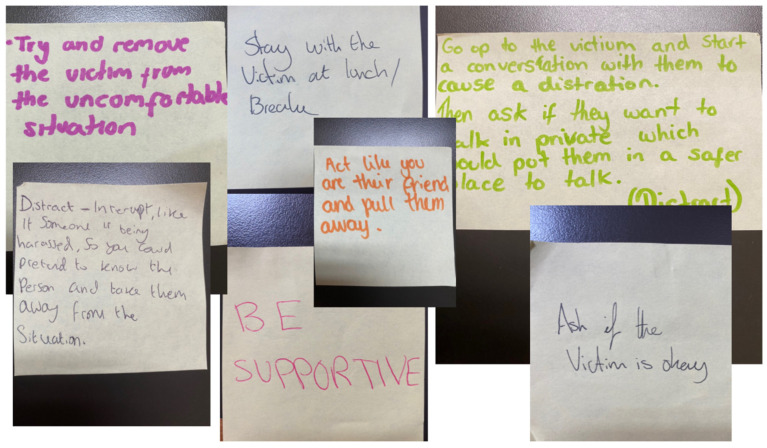
Examples of young people’s ‘Distract strategies’ (Hampshire, UK).

Rather than disciplinary measures of reporting and punishment, young people appreciated learning that they can respond to incidents of postdigital sexual violence by supporting the target after the incident. As seen in the post-it notes in Figure 4, many notes mention helping the victim through strategies like distracting the harasser, removing the victim from the situation, and making sure the victim is OK. Some young people came to feel they could talk about the issues more or use a range of strategies to help others:

Regina: I feel like what got into my head … was how you can just go up to them (target) and have a conversation with them afterwards even if you don’t know them. If I ever saw something happen, I think I might do that. I don’t think I ever really thought about that before, that maybe just asking them if they’re all right could help them.

(Cornwall, UK)

Helena: Yeah, a lot of the time it’s more like you’re trying to stop the perpetration. You kind of forget that maybe the victim needs support, and you say it’s not ok for the person to do that, and if you need help, I’m here.

(North Dublin, Ireland)

Roisín: If I saw a situation now, I’d certainly go over to them and ask if they were ok, did they need anything.

(South Dublin, Ireland)

Young people also talked about how discussing consent changed their understandings about how it relates to images, enabling them to think what might be possible in digital spaces like group chats:

Jay: Say if you’re in a group chat and then … like your friend had a boyfriend or something, then you guys break up, and then after she’s just sharing his nudes. But then I feel like the friends should speak up about it and say stop doing these type of things, or stop leaking it because she doesn’t have his consent to share with other people.

(Lewisham, London, UK)

Participants emphasised how our workshops allowed them to think critically about what they were witnessing online around nudes. Jay applies the content of the workshop about non-consensual sharing of nudes (Figure 2). Interestingly, he swaps the gender to create a scenario about a girl sharing a boys’ nudes. What is apparent is how the young people identify when they are witnessing abuse and violence online and thinking about the dynamics of consent in image sharing, and that it is important to ‘speak up’ and ‘stop leaking’.

### 3.2. Context Matters: Intersectional Challenges of Postdigital Bystanding

Across the study, however, young people felt school and community settings presented numerous challenges to bystanding due to various factors. Working across a wide range of schools, we were able to see how diverse school settings and distinct socio-economic contexts, including ‘inner city’ vs. ‘suburban’ or rural schools, created different power dynamics, which in turn shaped responses to the workshops.

#### 3.2.1. Sexism

For example, in a rural school in a farming community in East England, there was a notable “lad culture,” a British term which refers to a form of homosocial masculinity in group dynamics, which normalises sexism through humour and banter (Kehily & Nayak, 1997). It was not only the male students who cultivated the sexist lad culture as, female teachers explained that the male PE (physical education) and sport coaches at the school held the authority positions of Head Teacher and disciplinary lead (in charge of punishments), noting both men displayed overt sexism and were dismissive about issues of sexual violence and harassment at the school. Storer et al. (2017) argue that gender inequitable schooling environments present significant barriers to challenging sexual violence in these contexts. Indeed, in this school, the staff treated sexual violence among peers that happened off-school premises, including on social media spaces, as ‘non-school business’:

Tamara: A girl went and posted on the *Everyone’s Invited* website that two students from [our school] sexually assaulted her … and a couple of weeks before that, some boys made a virgin list, and I literally talked to him [staff member] about it. He said that boys at this age will be stupid, and that in year 9, 10, and 11, you won’t have to deal with this. I’m currently in year 9, and I still deal with this stuff.

(Norfolk, England)

In this example, the girls discussed reports made about the school to a popular website, *Everyone’s Invited*, which was founded in 2020 to expose rape culture in British schools. Tamara explains that the online testimonial was minimised by the Head Teacher.

This same overtly condoned sexism emerged in another school site in a small village setting in the West of England:

Heather: There’s a lot of the time where you speak to a teacher and they’re just like, ‘oh, it’s just boys being boys’, or something like that or, ‘just leave them be, you’re exaggerating’.

Rosie: Yes, my group told me specifically that, no matter what they put on the [*Everyone’s Invited*] list, the school won’t act on anything. So, there’s virtually no point to doing it.

(Cornwall, UK)

The young people felt the school’s sexism and lack of awareness of gendered power imbalances stemmed from a lack of up-to-date comprehensive sex education. They expressed concern that their schools do not cover sexual violence in relationship and sex education (RSE) and fail to discuss factors leading to sexual assault.

This was seen again in this focus group discussion in another semi-rural community with high levels of deprivation and a strong working-class ethos in the South of England:

Carey: I think we should have a lot more of personal and social health lessons as well. We only have one every two weeks, so we really don’t manage to cover a lot through the whole year. And it’s predominantly talking about straight sex always … it’s not inclusive at all.

Jane: More needs to be done when things do happen. Because they do happen, and not a lot comes from it, and they always tend to do it again. It’s normally the same sort of people that do it over and over again … if we had more lessons…the people who are doing it would be more educated and then probably would be less likely to do it.

Becca: This is because of probably safeguarding reasons for the teachers, but when they talk about sexual assault and violence, they speak as if the victim … like something external has assaulted them. They don’t put any blame on the assaulter, there’s no focus on the assaulter.

(Hampshire, UK)

The young people in this school complained about teachers lacking sensitivity and inclusivity in sex education, and in their understandings about sexual violence, for example, recounting an assembly where rules about skirt length were discussed on the same slide as sexual harassment (observation notes). The girls felt this was ‘slut shaming’ and ‘victim blaming’, and that this undermined any helpful discussion of sexual violence.

On the other hand, the boys at this school were concerned that the school was too punitive and blew things up:

Roderick: They deal with it wrong.

Joe: Yes. They don’t do as much, at least that’s what I’ve heard.

Interviewer: Can you just give us an example?

DeMarcus: They might just be like, really angry with it. Not really calming.

Interviewer: Angered. So they might make things worse, or …?

David: They just might punish someone, but you might not want them to punish someone, because if they find out you snitched they could do something to you.

(Hampshire, UK)

The boys discuss the perils of being seen as a ‘snitch’, which could create further risk and harm for the victim. These types of scenarios came up several times during our visits to this school. For instance, when an abuser and the victim were brought into a room to go over the reported image-based sexual abuse in an incident in which the girls’ nudes had been non-consensually shared, the victim found the experience re-traumatising rather than supportive, suggesting that the safeguarding procedures at the school were not working well.

#### 3.2.2. Masculine Defensiveness

A different set of contextual factors around masculinity emerged in an all-boys school in a wealthy area of Hertfordshire in England. Here, the boys seemed to particularly struggle with bystanding discussions which they felt were targeting them as ‘bad’ and ‘toxic’, and they became defensive:

William: I feel like all over in the media, just men are always portrayed as bad. Because if you go on social media … if I’m going through stories, I wouldn’t be surprised, maybe once or twice a day, I probably see something that points out how men are bad. And it’s like, it’s not a nice thing to see, being a male.

John: Grouping up every man into that one category, that everyone is a piece of rubbish.

William: Yes.

Rupert: Feel like attention is on us being bad [overtalking].

Samir: Like … we were at fault.

(Hertfordshire, UK)

While two of these boys suggest that bystanding is related to ‘being a gentleman’ and turning away from ‘toxic masculinity’, they also feel defensive about how attention to sexual violence is painting men as ‘rubbish’ and ‘bad’, which complicates their reception to sexual violence prevention work. They suggested that the workshop needed to better in showing something affirmative:

Harry: Think not getting rid of masculinity as a whole but displaying the positive masculinity.

Rupert: Yes. And I suppose being part of it is being a gentleman, and that comes to being an active bystander, it is part of masculinity. So, I suppose accepting masculinity and not turning it into a toxic thing but turning it into a good thing.

(Hertfordshire, UK)

The limitation of an all boys’ environment was also commented upon in another group:

Jake: It’s an all-boys school, later on we’re going to leave school, and if we haven’t been educated in school about problems outside of school, we’re never going to learn. And it doesn’t even matter if it’s going on at this school at this time. Even when we are out of school, we go home, or something, if you’re taking a bus and you see somebody feeling uncomfortable and you don’t know how to deal with it, there’s nothing you can do. So, the workshop would actually really help them on other stuff, apart from school.

Neil: Well, when you’re younger you don’t really want to talk about it because you don’t want to seem weak and there’s always being called a snitch and stuff, so there’s always that in the back of your mind to not want to say anything.

(Hertfordshire, UK)

The boys were aware of the barriers to enacting bystander practises both outside of their school context if they witnessed harassment of girls at home (so online) and in public (taking a bus), noting ‘they don’t know what to do’, but that the workshop could help them with strategies for that. However, the idea of being able to intervene in the homosocial masculinity peer group and stand up to other boys at school in this all boys’ setting was viewed as particularly daunting. Similar to the previous discussion of snitch culture, the boys’ fear of seeming ‘weak’ and putting one’s relationship with other boys at risk, often leaves them unwilling to say anything to other boys. This contradicts the discussion of being a ‘gentleman’ above and shores up some of the complexities of discussing active postdigital bystanding around sexual violence in all boys’ schooling environments (see also, (Ging et al., 2024)).

#### 3.2.3. Elitism: Opting Out

In both the Irish and Canadian contexts, we faced related challenges in two fee-paying schools, since attendance at the workshops was optional and required youth to obtain parental consent to opt into the workshops. This meant different school-based barriers emerged related to ‘choice’ and lack of attendance at the workshops, which were mediated through parental and student willingness to participate.

In Ireland, the team worked in a comprehensive school in which workshops were part of a regular RSE class. While students could opt out via a form signed by parents, no students did so, and the gender balance was even. In the fee-paying school, 60 young people participated, with only 12 boys signing up. The girls in the group discussed how boys wouldn’t sign up for the workshop because it was ‘uncool’ and there was no incentive to do so. As one student said:

Colleen: Yeah, it’s not a thing that people in our year especially would sign up for. Because they’d go Ugh! Not bothered.

(North Dublin Ireland)

The girls explained that they wanted to participate because they were interested in the topic and familiar with the issues discussed. They commented that girls are ‘closer’ to these issues and boys may avoid this type of workshop because they would feel ‘judged’. One of only two boys in one focus group acknowledged he only attended the workshop at the last minute because a female classmate reminded him it was happening, but he was ‘really late’ missing part of the session. It also emerged that many students had come to the secondary school from single-sex schools, carrying deep discomfort around working in mixed groups. One girl who had attended an all-girl school prior to the co-educational elite setting said she found doing sex education lessons in a mixed gendered group, which included labelling girls’ and boys’ ‘parts’, acutely ‘embarrassing’.

In the Canadian context, we worked in an independent private school, and the workshop was not part of the compulsory sex education curriculum. Recruitment was universal, with all students’ parents being sent consent forms, enabling many to opt out of participating. Consent forms were sent to 320 parents, and 108 students participated. Of these, 103 completed the pre- and post-workshop surveys: 51 females (50%), 35 males (34%), 15 participants who identified as non-binary or preferred not to reveal their gender (15%), and 2 (2%) who reported ‘other’. Thus, fewer boys participated in the workshops. Young people told us some of the parents were resistant and therefore would not give consent for participation:

Marie: I think a lot of parents were hesitant about it, because they see the word sexual harassment and … think … you’re going to be showing us, say, graphic images, or like something like that. Especially since some parents … their first language is not English. They might be confused or misled about some of the topics you are advertising. So, I think [instead of] sexual harassment online it could be like media or digital citizenship, or something sort of being like a lot more upfront about what would be included in the workshop. I feel like more people should have attended. I think it should have been less of a voluntary thing, and more of a mandatory thing… I think it would have been a lot better if a lot more people went because it’s a really important issue and topic.

(Ontario, Canada)

Another student concurred that the low attendance was sometimes because parents refused, but also in some cases because students just did not want to participate.

Kate: So, like everyone who went was part of two gym classes in total … In the gym class, would probably be around 50 people, but the people that attended the workshop was actually only 20, and it was a very similar demographic. So, it was people who generally were, probably knew more about sexual harassment, or more open to the idea of learning about it…A lot of people I talk to after the workshop they found the same …. (1) They wanted to go, but their parents did not let them; or (2) They were mostly … male aligned people … a lot of them were not there, and … in our grade …there’s talk about a certain group being kind of more … ignorant about the problem.

(Ontario, Canada)

This participant noted that ‘more ignorant’ ‘male aligned’ students refused to participate, and the workshops only attracted a particular demographic. She went on to say that as part of that group who ‘knew more about sexual harassment’, they needed more support on how to respond to the issues raised:

Kate: As for the actual workshop itself. I feel I became more clear with the specific terms, like … sexual abuse and online sexual harassment. However, I feel like a lot of the knowledge … I already knew and like for the knowledge I didn’t know—for example, specifics of what to do if you are sexually harassed—I still don’t.

(Ontario, Canada)

This participant felt that concrete steps for the school to support bystanding were not made evident, and this points to a need to change the ‘whole school’ environment to address sexual violence.

#### 3.2.4. Racism

One school site in Southwark London was located in a highly diverse area with a majority Black British population. The rich discussions in the workshops covered their explanations of racism in their everyday life, including mentioning historical examples of racism to understand bystanding, as one boy noted in the workshop “Rosa Parks and was made to sit at the back of the bus. Who are the bystanders—the white people in the bus” (observation notes). The young people were also rightly concerned about the “document” strategy of filming violence to report it, which they felt could be very risky, with another boy noting in the workshop “If you pull a camera people can get aggressive and sabotage you. People doing something bad wouldn’t want to be recorded” (observation notes). In the interviews, they further noted that any type of confrontational technique would be highly risky and unrealistic in public space or on public transportation, which many use to and from school:

Dionne: I think that they’re helpful but for certain types of people. If you’re an active bystander but you don’t like confrontation, it’s not realistic. … Let’s say you’re on a bus or a train or a Tube and you see something happening, you’re way less likely to speak up about it. And the most realistic one I saw was ‘delay’ and I didn’t even think that was very helpful. It does cater to the victim, but you’ve kind of enabled it because you haven’t stopped it. I think the five Ds, they give you information about ways you could intervene or stop it in a sense, but how likely is it that people are actually going to do that? Because a lot of the culture is to not look, pretend you didn’t see anything.

Mia: Or ignore it when something’s happening … the five Ds didn’t show anything that people who would rather not directly involve themselves, especially with the aggressor, it didn’t show what we could do basically.

Clarice: I think ‘distracting’ can possibly put your safety at risk if the harasser, he then puts you in the same book as the victim and starts harassing you.

Dionne: But I’d want someone to help me, but I also wouldn’t want to put someone else in danger at the same time. People are unpredictable.

(Southwark London)

In these examples the girls are referencing ‘certain types of people’ who perhaps have more power and privilege than young Black Londoners to intervene than is safe for these youth when they are out in public in London. They also discussed barriers in their school context where confrontation is too risky and it was better ‘not to get involved because you can get hurt’:

Jamar: Let’s say there’s a group of people bullying someone and then obviously they’re probably the popular people, so you wouldn’t want to help them, even though you’d feel bad. Because you don’t want it to flip on you and they start making fun of you and doing all of this and …

Aaron: Yes, and it’s like next time I should mind my business …

Caleb: I feel like there should have been more about what you could do in that situation because the five Ds, as they were helpful, they didn’t give you enough choices … Too many of them were confrontational.

Jamar: Yes, and most of us don’t like confrontation but still want to help.

Caleb: sounds quite unrealistic and unlikely.

(Southwark London)

In both extracts, we can see the girls and boys are fatalistic, feeling bystanding will not work for them, which gels with Storer et al.’s (2017) findings about ‘pessimism’ regarding these tactics. Our point, however, is that their daily context shapes this pessimism. The young people repeatedly discuss wanting to help, but feeling like it is not possible because of structural factors which make it difficult to intervene in conflicts in their school amongst their peer groups, since confrontation could easily escalate into further violence. They express feeling conflicted about these strategies as a result.

#### 3.2.5. Reluctance to Report Digital Issues

In relation to digital issues in particular, the young people noted a strong reticence to bring issues to the attention of teachers because they knew teachers could not keep such matters confidential and would have to involve parents:

Donte: I think they call your parents and have a talk about it. In case something gets leaked across social media platforms then you need to, because you know how we’re young, it’s classified as child pornography, so they need to call your parents and so ….

(Southwark London)

According to the young people, online episodes are treated as ‘child pornography’, which makes it unsafe for young people to bring these issues to school attention, because they will swiftly be escalated in ways that will create more difficulty for students. This problem of categorising youth sexting as child pornography has been explored internationally, demonstrating that finer distinctions need to be made in legal and school-based responses (Setty, 2023) to youth-produced intimate images. They went on to strongly disagree that they could report these types of digital sexual violence at school:

Interviewer: Would you feel comfortable going to your school if something like that [nudes being shared online] happened to you?

Sander: No.

Calvin. No

Halle: No, no, no, no.

Interviewer: Why?

Halle: Because I feel like they will just judge you … They’ll just think that’s your fault. Say if something happened in school between two people, and then if the girl didn’t like what happened they will exclude the other person but they will still … Like if you’re the victim of harassment, you feel punished.

(Southwark London)

These participants were adamant they would not report digital issues around sexual violence to their school. Three participants say “No”, and Halle repeated “No” four times to underscore how avidly she disagreed. The reason Halle said she would not tell anyone at school is because she felt the school will blame the victim, in this case the girl, and even if the boy was excluded (suspended), the issues will continue to impact the girl. As with earlier schools, Halle felt the victim of sexual violence would be the one to suffer. As she put it, ‘like why are you being punished if you’re the one that received the harassment, if that makes any sense’. (Southwark London).

Another group explained that they were concerned the school would escalate the situation beyond parents to the police:

Noah: They [the student] would probably only want it to be with the teacher and maybe they figure out from there, but then the teacher will probably…get in contact with the police or something like that. And then the police will get involved and then their parents will get involved, and then too many people will be involved, and it will be a really big situation. That’s why sometimes they just keep it to themselves because they don’t want it to go to extreme measures.

(Southwark London)

The students portray a situation in which there are disproportionate responses to digital issues, with schools consistently involving the police and leading to what the young person above calls ‘extreme measures’. Given statistics on disproportionate criminalisation of black boys and young men in England, these concerns are warranted (Whitehead & Ringrose, 2021).

### 3.3. Post-It Notes for Change: Capturing Young People’s Ideas for Making Schools Safer Places

**Figure 5:**
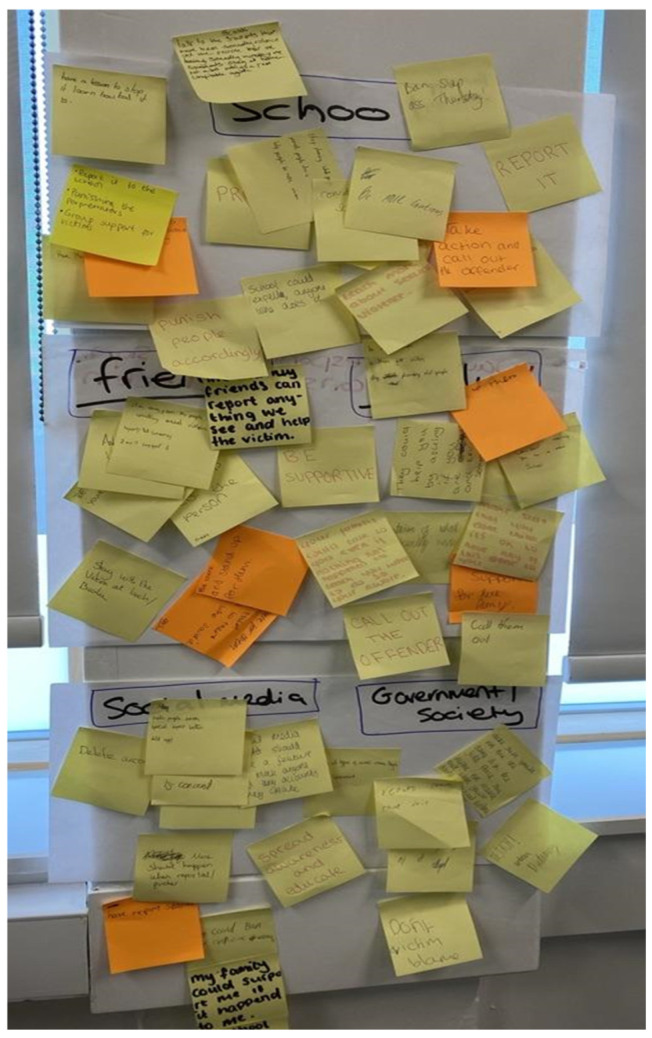
‘How can we challenge sexual and gender based violence’ post-it note activity (Hampshire, England).

In the final analysis section, we would like to build upon the ideas of what needs to change, as communicated by the young people in one of the group activities in Workshop Two, in which we asked young people to think about how family, government, school, and social media could improve their responses to sexual violence. Here, we focus on how young people think schools need to change. Young people worked in their groups either together or separately, writing their messages for change onto post-it notes. They were then asked to post their messages in the appropriate category sheets of paper hung around the room (see Figure 5).

These post-it notes enable the expression of thoughts and feelings during the activity in ways different from interviewing methodologies after the fact, capturing a more live youth experience (Renold et al., 2024). Many participants found this activity beneficial. For example, according to Becca (Hampshire, England), the post-it notes activity enabled anonymity regarding the issues discussed and helped students to be more ‘responsible’ and ‘mature’, thereby mitigating against the associated anxiety and banter of large group discussions about gender-based violence.

#### School-Based Change

Young people’s calls for school-based change can be grouped into three broad interrelated themes: ‘Make school a safer place’, ‘Listen and take action’, and ‘Educate & raise awareness’. These key themes can be considered the groundwork that would make postdigital bystander work more impactful.

**Figure 6:**
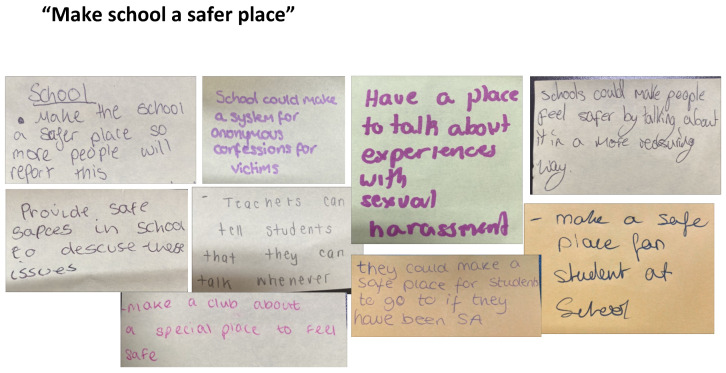
Post-it notes for making schools a safer place.

The messages in Figure 6 indicate that, as a first step, the young people in our study want a place to talk about these experiences, including online sexual harassment. The school needs to create a ‘safe space’ or ‘place’ where that can happen. We are mindful that ‘safe space’ is a misnomer and no space can be 100% safe. It is critical, however, to go beyond ‘safeguarding lip service’ in relation to sexual violence in schools and online (Ringrose et al., 2021). If schools are shutting down conversations about postdigital sexual violence and ways to respond like those featured in our workshops, then safeguarding is not working because young people are simply refusing to talk about it. As one student writes, ‘make the school a safer place to more people will report this’. Another interesting response by one student is the suggestion to create a space for ‘anonymous confession’, which appears to highlight that those reporting and discussing sexual violence need a space where confidentiality is enabled and in which escalation would not occur.

**Figure 7:**
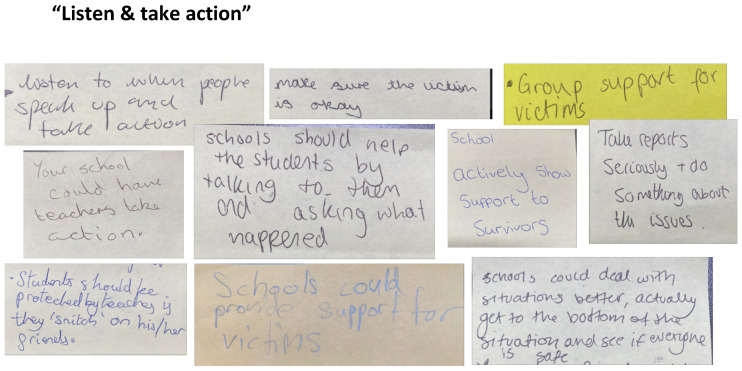
Post-it notes for listening and taking action.

In the comments in Figure 7, schools are urged to be more responsive to young people through listening and offering support. The word support appears in three of the post-it notes, showing that young people want schools to respond to issues of sexual violence and to take these issues seriously, provide help and ‘protection’ for victims, and ‘actively show support for survivors’, which incorporates bystander principles at an institutional and structural level in the school.

**Figure 8:**
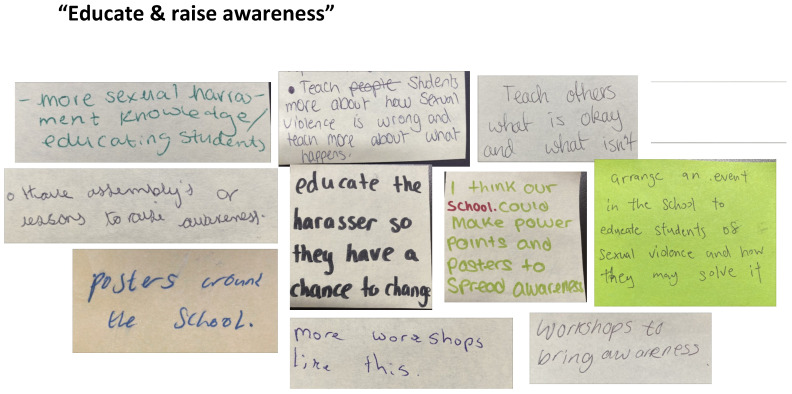
Post-it notes for education and raising awareness.

Tied to the previous themes of young people asking for more safety and for better listening, responsiveness, and support, the final theme is students wanting schools to be proactive and educate, Figure 8 on these topics. This responds to the discussions about the weaknesses and gaps in their sex education and sexual violence prevention procedures at school. The young people had suggestions about how schools need to create environments more like the workshops we delivered to educate on these topics. One student suggests that schools need to ‘educate the harasser’. This corresponds to research on girls’ experiences of being the focal point of gender and sexual violence awareness training, rather than cis gendered and heterosexual boys and masculinity practices (Keddie, 2022). There was a mention, as there was in the focus groups, of a need to discuss the different categories of violence, including sexual harassment, through teaching, events, assemblies, posters, and workshops like the ones we delivered to ‘spread awareness’. The young people are in effect advocating for a whole school approach to addressing sexual violence to provide a context in which postdigital bystanding practises could be possible, safe, and practically work (EVAW, 2023).

## 4. Discussion

Across the three countries, we found major gaps in school provision of sex education, with more than one-half of young people in every context noting they had not previously learned about sexual violence. Our active bystander workshop introduced young people to strategies to intervene into contexts of postdigital sexual violence both at school and online. The improvement in understandings of bystanding was variable, but significant across all countries, with a 27% increase in Canada, 33% in Ireland, and 40% in England.

Despite increased understandings, however, the realities of the contexts of their postdigital lives made the bystander interventions difficult to put into practice. Young people described not being comfortable, reporting episodes of violence at school due to gender-inequitable environments that condoned sexism, sexual violence, and harassment in ways that aligned with Storer et al.’s (2017) study. We found lack of understanding about sexual violence from some teachers and ineffective school responses in some cases. In one school, the head teacher dismissed episodes of sexual violence at the school which were reported on the website *Everyone’s Invited*, with a pat response ‘boys are stupid’. In another school setting in England, the young people complained that their teachers did not understand sexual violence, noting their PSHE (personal, social, and health education) lead delivered victim-blaming slut-shaming assemblies about girls’ inappropriate skirt lengths; at this same school, they also discussed an example where a victim of image-based abuse was brought into the same room with the perpetrator at school to go over the incident, which was found to be retraumatizing.

We found particular issues emerged in the context of the sheltered all-boys school, with some boys reacting defensively in response to bystanding, while others noting the challenges in confronting peers. This aligns with Harder’s (2020) research that men and boys face significant challenges and ambivalent feelings around confronting dominant masculinity practices via bystanding practices. In the fee-paying schools, many boys opted out of the workshops altogether, rejecting the chance to learn about sexual violence and bystanding when given the opportunity. There was much greater attendance by girls, one of whom in the Canadian case complained that the workshops became a case of telling girls what they already knew about sexual harassment, but that because boys did not participate, she still felt ill-equipped to challenge sexual harassment in her school.

Our young Black British participants additionally explained contexts of racism, which made it risky and unrealistic for them to practice bystanding techniques in public spaces like transport or at school. They felt ambivalence and contradiction again, as with Harder’s (2020) research, and a lack of realism in bystanding tactics, as with Storer et al.’s (2017) study saying they understood that they should intervene but that the social, cultural, and schooling contexts prohibited them from doing so. They also discussed disproportionate responses to digital issues, including the school categorising leaked images as child pornography rather than recognising image-based abuse. The young people discussed teachers going too quickly to parents and police, which meant they were afraid of discussing digital dynamics and conflicts at school. Our participant, Halle, explained that, in relation to the non-consensual sharing of nudes, that the girl would be judged. In ways that seemed to reflect how rape culture plays out in wider culture, Halle explained that the victims of sexual harassment end up feeling ‘punished’. Here, we draw attention to the felt experiences of school responses, which are experienced as punitive rather than supportive. We see that ‘ineffective school responses’ (Storer et al., 2017) present a major barrier to active bystanding, since schools typically could not promise young people confidentiality due to safeguarding policies, and particularly those around digital disclosures, and processes of penalising perpetrators could escalate situations and sometimes bring further risk and harm to the victims.

The final section explored ways of addressing issues of barriers to bystanding in school spaces, drawing upon young people’s post-it notes for changes they wanted to see from schools. This creative methodology offered a unique way to grapple with youth voices. Grouped into three themes, we saw first that young people wanted schools to be ‘safer’ for discussions of sexual violence. Second, they wanted schools to listen and take action to actively support victims and survivors of sexual violence, in counterpoint to the types of punitive approaches discussed above. Finally, young people wanted schools to continue to raise awareness, particularly to educate the ‘harasser’, which relates to the urgent need to prioritise boys and men in postdigital bystanding work rather than creating contexts where some boys can dwell in defensiveness or simply opt out of such education altogether. It is interesting that although the discussion of sexual violence related to what happens online and offline in schooling environments, the discussion of what schools could do mostly focused on providing supportive and safe spaces, rather than the school intervening into the digital dynamics, involving the police, parents, or other tactics. The key overall message drawn from these themes is that young people want schools to provide support and safety for them to learn about these issues.

## 5. Conclusions

In this paper, we argue that young people are operating in postdigital contexts where online and offline sexual violence are entangled, and these dynamics must be considered in any educational interventions that attempt to intervene into such contexts and to upskill and empower bystanders. We introduced the concept of postdigital bystanding to acknowledge the more-than-digital complexities of peer social relations and school life, since what happens online often travels and follows young people back into their school settings and schools are left having to try to manage and support young people dealing with postdigital dynamics on an everyday basis. We argued that this attention to postdigitality has not yet been addressed in the bystanding intervention research, and that our conceptualisation offers some unique insights into designing and implementing sexual violence interventions in school sites.

Overall, by drawing upon a range of creative methodologies to better understand youth responses to such educational interventions, our research underscores the need to take seriously and listen to young people’s ideas about how we can better support them. It follows that our main recommendation is to listen to youth. The young people’s key messages were simply to provide safety and support around discussing these issues, as was enabled in our workshops. We take heart from the young people’s voices and have sought to bring them into view so that sexual violence interventions and bystander work in schools may have a greater chance of enabling genuine social change.

## Data Availability

The data presented in the study are available on request from the corresponding author.
